# Health information systems and tackling violence against women: a necessary dialogue

**DOI:** 10.1590/1980-220X-REEUSP-2024-0353en

**Published:** 2025-06-13

**Authors:** Alice Cristina Medeiros Melo, Isabella Vitral Pinto, Camila Alves Bahia, Carmen Sant Fruchtman, Taynãna César Simões, Daniel Cobos, Letícia de Oliveira Cardoso, Paula Dias Bevilacqua

**Affiliations:** 1 Ministério da Saúde, Secretaria de Vigilância em Saúde e Ambiente, Departamento de Análise Epidemiológica e Vigilância de Doenças Não Transmissíveis, Brasília, DF, Brazil.; 2 Fundação Oswaldo Cruz, Instituto René Rachou, Grupo de Pesquisa Violências, Gênero e Saúde. Belo Horizonte, MG, Brazil.; 3 Universität Basel, Schweizerisches Tropen- und Public Health-Institut, Allschwil, Schweiz.; 4 Fundação Oswaldo Cruz, Instituto René Rachou, Núcleo de Estudos em Saúde Pública e Envelhecimento. Belo Horizonte, MG, Brazil.

**Keywords:** Database, Public Health Surveillance, Women's Rights, Comprehensive Health Care.

## Abstract

**Objective:**

To identify information obtained from Health Information Systems (HIS) allowing to detect violence against women, critically discussing strategies to promote the integration of these data and the improvement of analyses on violence.

**Method:**

Reflective theoretical essay, using a narrative review, based on the analysis of national documents on five HIS and their potential for detecting violence against women. The article discusses strategies developed within health management and academia to promote the integration and availability of data and information on violence against women.

**Results:**

The five HIS described (SIM, SINAN, SINASC, SIH and SIA) present possibilities for building diagnoses and tackling violence against women that can be better exploited by health surveillance.

**Final considerations:**

The integration of databases to produce information is strategic for tackling violence against women, enabling cases to be monitored and women to be offered more comprehensive and humanized care, helping to prevent extreme events such as femicide.

## INTRODUCTION

Health Information Systems (HIS) are indispensable tools for decision-making, planning, monitoring and evaluating health situations and actions^(1)^. In Brazil, the development of nationwide HIS has followed the progress of public health policies and the specific needs of different areas within the Ministry of Health (MoH), as well as the evolution of information technology^(2,3)^.

The HIS in operation in Brazil play a crucial role in providing clinical and care information, as well as essential data for formulating and monitoring morbidity and mortality indicators. These systems are valuable sources of evidence for analyses of the health situation, and are fundamental for conducting studies aimed at characterizing and epidemiologically analysing diseases and other health problems^(4)^.

The use of HIS to analyze the situation of violence gained visibility in 2001, with the publication of the National Policy for Reducing Morbidity and Mortality from Accidents and Violence^(5)^, and in 2006, with the creation of the Violence and Accident Surveillance System (VIVA in the Portuguese acronym). These milestones were added to other actions promoted by the Brazilian government in response to the need to develop policies to tackle violence within the health sector, highlighting the role of HIS in monitoring and preventing this problem^(6)^. Specifically in the field of gender-based violence, the National Policy to Combat Violence against Women^(7)^ considers the production and systematization of data and information on violence to be a priority, and these activities are carried out by multiple sectors such as health, public security, justice and social assistance.

A study based on documentary analysis identified the existence of 54 different national HIS in operation between 2010 and 2018, under the realm of the Ministry of Health^(2)^, as well as state and municipal initiative systems designed to meet local specificities and needs. Girls and women access health services for routine or emergency care. As violence has consequences for physical, mental, reproductive and/or sexual health, and can also affect the management of chronic diseases^(8)^, it is expected that women in situations of violence will seek health services more often^(9)^. It is therefore essential that these services have the capacity to detect cases and record them in the information systems available at each level of care. However, it is important to note that despite the variety of information systems available, information on women and events related to violence is often presented in a fragmented way in the systems^(10)^. In addition, the systems operate independently of each other and there is no sharing of data and information between them^(11,12)^. This fragmentation limits a comprehensive and longitudinal understanding of the phenomenon, hindering decision-making and action within the scope of health surveillance and care.

The Brazilian scientific literature is rich in articles that analyze the characteristics of violence and individuals in situations of violence, based mainly on notification and death data^(13)^. However, there are few studies discussing the contributions of HIS to monitoring violence against women, based on an integrated and longitudinal approach to systems and their capacity to respond. Among the studies identified, we would mention Okabe et al.^(10)^ and Carvalho et al.^(14)^, the first of which makes individualized analyses of Notifiable Diseases Information System (SINAN in the Portuguese acronym), Hospital Information System (SIH in the Portuguese acronym), and Mortality Information System (SIM in the Portuguese acronym), i.e. it discusses the contributions of each of the systems to monitoring and decision making for tackling violence. The second is an integrative review using 25 studies carried out in Brazil and other countries, with the studies selected in Brazil analyzing SINAN data in particular. Although they make contributions, these studies do not address the challenges and strategies for integrating data, and there is a need for constant updating on the subject, since tackling violence against women continues to be a major challenge for health and other sectors, whether due to its magnitude, severity or the speed with which it produces new forms of violence.

Therefore, in this article, based on five national HIS (Live Birth Information System - SINASC; Notifiable Diseases Information System - SINAN; Hospital Information System - SIH; Outpatient Information System - SIA; and Mortality Information System - SIM), we update the discussion on the information that these HIS can provide on violence in general and, where relevant, on specific types of violence against women. In addition, we critically discuss strategies that have been adopted to promote the integration of these data and the improvement of analyses on violence against women through the integrated use of HIS. Considering the importance of this phenomenon, which has different magnitudes and produces different health impacts, as well as political, economic, social and cultural implications, whether for women survivors or for cases of extreme violence, such as femicide, it is important to have information systems that allow for a better understanding of cases of violence and consequent events, such as death.

As our methodological approach, we carried out a narrative literature review, based on a selection of scientific articles published in different journals and technical-scientific documents produced by the Ministry of Health. A narrative literature review is a method that uses a variety of information sources, but does not aim to exhaust the search for studies dealing with the subject of the review, as it does not use controlled data search procedures^(15)^. The choice of national publications in this study is justified by the fact that we are interested in reflecting on the Brazilian reality in terms of the capacity to produce information and inform public policies from existing health information systems.

## OPPORTUNITIES TO MONITOR VIOLENCE AGAINST WOMEN IN HEALTH INFORMATION SYSTEMS

Based on the chronology of life events, all births are recorded in the Live Birth Certificate (DNV in the Portuguese acronym), the basic document of the Live Birth Information System (SINASC). This system was created in 1990 with the aim of overcoming the problems of coverage of live birth registrations, which had previously been done in civil registry offices, standardizing data collection throughout the country, and expanding the data to allow for the construction and monitoring of indicators of the maternal and child health situation^(16)^ (Chart 1). SINASC provides demographic and epidemiological information on newborns, parturient, prenatal care and childbirth^(17,18)^, serving as the basis for calculating indicators such as fertility rate, birth rate, infant mortality rate and maternal death ratio^(19)^ (Chart 1).

**Chart 1:** Characteristics of Health Information Systems (HIS) – Belo Horizonte, MG, Brazil, 2024.

Characteristic	SINASC	SIA	SIH	SIM	SINAN
Year Launched	1990	1990s	1975	-	2006 (violence events)
Chronology of Monitored Events	Birth	Throughout life	Throughout life	Death	Throughout life
Registration Instrument	Live Birth Declaration (DN)	Consolidated Outpatient Production Bulletin (BPA-C); Individualized Outpatient Production Bulletin (BPA-I); Authorization of Outpatient Procedures (APAC)	–*	Medical Certificate of Cause of Death (MCCD)	Notification form for interpersonal or self-inflicted violence
Objective	Recording demographic and epidemiological data on the newborn, mother, prenatal, and childbirth	Control and monitoring of outpatient care and treatments	Registration and payment of hospitalizations in public and SUS-affiliated hospitals	Recording socioeconomic data of the individual, death characteristics, and reporting professional	Continuous surveillance of violence cases
Coverage	98.2%	–*	70% to 80% of total hospitalizations	96.7%	–*
Data Type	Individual	Aggregated and individualized	Individual	Individual	Individual
Monitoring of Violence Against Women	Pregnancies of girls under 14 years old	Registration of specific data on sexual violence (from 2014).Primary and secondary diagnoses based on ICD-10.Procedures carried out that can be associated with violence, e.g. injuries, trauma, depression, anxiety, exacerbation of chronic illnesses	Joint analysis of the primary and secondary diagnoses of the event that led to hospitalization.Primary diagnosis: data on the type of injury or trauma, according to Chapter XIX of ICD-10.Secondary diagnosis: characterization of the event, according to codes in Chapter XX of ICD-10, for example, whether the event is the result of an accident, interpersonal violence or self-harm.	Monitoring of femicides based on causes classified as assaults or homicides (ICD-10 codes X85 to Y09)	Direct registration of violence cases; suspected or confirmed cases of domestic, sexual, self-inflicted violence, human trafficking, slave and child labor, torture, legal intervention, and homophobic violence; records of extrafamilial or community violence against children, adolescents, women, elderly, disabled persons, indigenous people, and the LGBT population

Note: “-” indicates no specific data available.*

Although this system does not have a specific field for recording or reporting violence, information on the age of parturient women can reveal a situation of rape of a vulnerable person (Chart 1). According to the Penal Code, this crime is defined as the existence of carnal conjuncture or the practice of another libidinous act with a minor under the age of 14, with “the victim’s consent for the act, her previous sexual experience or the existence of a romantic relationship with the agent being irrelevant”^(20)^. Considering only the SINASC registry, the presumption (or detection) of sexual violence, followed by pregnancy resulting from violence, only occurs at the time of delivery.

Considering the situation of vulnerability of girls and adolescents, as well as the presumption of sexual violence, data from the Live Birth Certificate (DNV) offers the possibility of identifying various aspects, such as schooling, race/skin color, marital status, history of previous pregnancies, quality of prenatal care (including when it began and the number of appointments made), as well as information related to childbirth and the newborn (such as birth weight and Apgar score). In addition to the importance of these data for producing a comprehensive epidemiological characterization, they are also crucial for guiding the actions of primary health care, offering information for organizing comprehensive care for mothers and their babies. A study published in 2024 used SINASC data to analyze the spatial distribution of pregnancy in girls under 14 years of age, revealing greater vulnerability to sexual violence in residents of the North and Midwest regions of Brazil^(21)^.

SINASC coverage in Brazil was 98.2% in 2020, but there is great heterogeneity between municipalities, especially in the North and Northeast regions, which had the lowest coverage percentages, 95.5% and 96.5%, respectively^(19)^ (Chart 1).

Morbidities that occur throughout an individual’s life are recorded in different systems, such as the Outpatient Information System (SIA), the Hospital Information System (SIH) and the Notifiable Diseases Information System (SINAN).

The SIA was implemented in the 1990s with the aim of controlling and monitoring outpatient care and treatment, through the Management System for the List of Procedures of the SUS (SIGTAP/SUS in the Portuguese acronym). This system registers everything from basic care consultations to procedures carried out in emergency rooms and urgent/emergency outpatient clinics, in order to invoice health services provided by the SUS, thus defining the transfer of financial resources between the federal, state and municipal governments^(22)^ (Chart 1).

From the SIA we can access information in aggregate form (Consolidated Outpatient Production Bulletin/BPA-C) and individualized form (Individualized Outpatient Production Bulletin/BPA-I and Authorization of Outpatient Procedures/APAC)^(22)^. In 2014, the procedure “multiprofessional care for comprehensive attention to people in situations of sexual violence” was created in the SIGTAP table, which must be recorded in the BPA-I and includes sexual assault by means of physical force; sexual abuse; problems related to sexual abuse alleged by a child and practiced by a person within their family group; and problems related to sexual abuse alleged by a child and practiced by a person outside their family group^(23)^ (Chart 1).

In addition to this procedure, which specifically informs the situation of sexual violence, the primary and secondary diagnoses, recorded on the basis of the International Classification of Diseases - 10th Edition (ICD-10), as well as procedures carried out according to the demand for care, can be related to the effects (acute and chronic) of the violence (Chart 1). These effects include injuries, trauma, depression, anxiety and worsening of chronic illnesses, as shown in different studies^(8,24)^.

However, the authors of this study are unaware of any studies that use SIA data to evaluate procedures related to violence against women. In the 25 scientific papers selected in an integrative review, there was no mention or analysis of outpatient data to characterize cases of violence against women^(14)^.

In health events requiring hospitalization, the SIH is the reference system, used to record and pay for these occurrences in the public network (federal, state, municipal) and those affiliated with the SUS^(25)^, covering around 70% to 80% of all hospitalizations in the country^(26)^ (Chart 1).

From the SIH, we can obtain information on the main and secondary diagnosis based on ICD-10, as well as demographic and geographical information for each hospitalization, supporting the planning of health actions and the production of epidemiological data (hospital morbidity/mortality)^(25)^. The primary diagnosis refers to the main reason for hospitalization, while the secondary diagnosis refers to all the conditions that developed during hospitalization or that affected the care received and/or the length of stay in hospital^(27)^ (Chart 1).

Cases of violence in women’s hospitalization records can be identified from the primary diagnosis, which informs the types of injuries or trauma, according to chapter XIX of ICD-10^(28)^. However, the characterization of the event is based on the secondary diagnosis, filled in with the codes from Chapter XX of ICD 10^(28)^, which inform, for example, whether the event is the result of an accident, interpersonal violence or self-harm, and it is essential to analyze both diagnoses together in order to identify cases of violence.

Despite the importance of hospital data for characterizing and monitoring events of violence^(5,10)^ and the efforts made to improve the recording of primary and secondary diagnoses of hospitalizations^(10)^, it can be said that scientific production using SIH data is still timid compared to what is produced using mortality and notification data.

Death, as the last event in a person’s life, is recorded in the Mortality Information System (SIM) through the Medical Certificate of Cause of Death (MCCD). SIM, created in 1975, is the most consolidated system in Brazil, with coverage rates of 96.7% in 2020^(16)^ (Chart 1). However, coverage rates between Brazilian regions show significant heterogeneity: South (100%), Southeast (98.4%), Midwest (96.7%), Northeast (93.4%) and North (92.1%)^(19)^.

The MCCD is made up of fields that provide socio-economic data on the individual, the characteristics of the death and the informing professional. Based on the information contained in the MCCD, it is possible to calculate indicators such as the maternal mortality ratio, general and specific mortality rates (according to age groups, sex, causes of death) and the proportion of mortality by cause^(19)^. This system allows for the identification of extreme violence against women, i.e. violence that results in a fatal event (Chart 1).

Doctors are exclusively responsible for filling in the MCCD^(29)^, and in cases of death due to external causes, the MCCD is issued by doctors at the Medico-Legal Institutes, services linked to Public Security in the states.

In order to analyze deaths due to violence against women, i.e. femicide^1^, the causes classified as assaults or homicides (ICD-10 codes X85 to Y09) are generally selected^(30)^.

However, in the MCCD, consummated femicides cannot be differentiated from homicides, since there is no specific classification in the ICD-10 for this cause of death. In these cases, since femicide is a qualifier of homicide, these deaths end up being recorded with codes X85 to Y09. Some studies have made efforts to make femicide visible using SIM data, such as the Atlas of Violence, which, since the 2020 edition, has considered the event ‘homicide of women in the home’ as an indirect measure of cases of femicide^(31)^.

In addition, deaths of women classified as suicide, accidents or ill-defined causes of undetermined intent can hide situations of interpersonal violence that led to death. For this reason, the investigation of women’s deaths is important, requiring specific analysis of these records, or even linking them to public security data in order to properly qualify the death.

In the case of violence against women, the Notifiable Diseases Information System (SINAN) is the system in which this event is recorded directly and systematically, using the Individual Notification Form. Violence surveillance was implemented by the Ministry of Health in 2006 through the Violence and Accident Surveillance System (VIVA), which consists of two components: sentinel surveillance and continuous surveillance^(32)^. Continuous surveillance became compulsory in 2011, when violence was included in the list of notifiable diseases^(33)^ and must be recorded in SINAN by health professionals in public and private services. Any suspected or confirmed cases of domestic/intrafamily violence, sexual violence, self-inflicted violence, human trafficking, slave and child labor, torture, legal intervention and homophobic violence against women and men of all ages must be reported. In the case of extra-family/community violence, only violence against children, adolescents, women, the elderly, people with disabilities, indigenous people and the LGBT population^(32)^ will be reported (Chart 1).

SINAN has filled a gap in the health sector’s recording of violence, since the case is recorded directly and not as a consequence of an event, such as care, hospitalization or death.

Since violence was incorporated as a compulsory notifiable disease, SINAN data has been widely used to analyze and characterize individuals and the different events related to violence against women. Despite efforts to consolidate and expand reporting throughout the country^(6)^, underreporting is still a challenge for health services in all of Brazil’s federal units^(34)^.

Considering the characteristics of the HIS presented, it can be seen that these systems were designed and organized to meet specific demands, such as, for example, the recording of an event/injury or procedure, and ways of working that are specific to the SUS. Despite the limitations in terms of coverage, completeness and data quality, as well as the lack of interoperability between the systems, their data has been continuously used to produce diagnoses related to violence against women. In this sense, they have played a fundamental role in making the problem visible and in planning policies to improve actions to combat violence.

## THE ROLE OF *LINKAGE* IN SEARCHING FOR AND DETECTING VIOLENCE AGAINST WOMEN

Given that HIS are designed and structured for specific purposes, whether for surveillance, care or health management, often independently, redundancy, discrepancy or complementarity can be found in the data between different systems. While on the one hand this diversity of information is beneficial, on the other it requires investment in integrated analysis involving the various databases.

Due to the absence of a unique identifier and the lack of interoperability between the national HIS^(35)^ discussed in this article, research and management areas have resorted to *record linkage* as a strategy for integrating information and obtaining a more comprehensive view of cases of violence against women. The use of record linkage techniques can help to identify patterns, gain a deeper understanding of the dynamics of violence and provide important evidence for the development of effective policies and interventions.

*Linkage* can be defined as a method of searching for identical pairs in the same or different databases^(36,37)^. This technique makes it possible to find pairs of records referring to the same person, as well as improving the completeness and consistency of the data^(36,37)^. The absence of a unique and consistent identifier, which makes it possible to locate the same individual in different databases^(4)^, such as the Individual Taxpayer Registry (CPF in the Portuguese acronym), for example, implies greater complexity, requiring the use of two or more key variables, such as name and date of birth. However, in these fields there may be typing errors, the use of abbreviations and missing values, which will affect the quality of the final *linkage* result^(35,38)^. In the case of the deterministic approach, in which there needs to be total agreement for the pairs to be formed, small differences in names or date of birth would result in the records not being matched and a consequent loss of true pairs^(37)^. In the probabilistic approach, it is necessary to determine a cut-off point for assessing the similarity of pairs, always seeking to reduce false positives and false negatives.

Several studies have used *linkage* techniques to understand the repercussions of violence on women’s lives, increase case detection and assess the risk of avoidable outcomes. In the United States, Cerel et al.^(39)^ used *linkage* to analyze the relationship between violent deaths and previous admissions to emergency services due to injuries from external causes. The technique was also used to locate maternal deaths that were associated with violent deaths in North Carolina/USA^(40)^. In Brazil, *linkage* has already been used to analyze deaths due to aggression^(41)^, sexual violence and its subsequent repercussions on pregnancy and childbirth^(42)^, women with notification of intimate partner violence and death from any underlying cause^(43)^, risk of death in women with notification of interpersonal violence^(44)^ and mortality in women with notification of violence durin^g^ pregnancy^(45)^.

The integration of health information systems is also considered an important strategy of the National Health Surveillance Policy (PNVS in the Portuguese acronym), in order to contribute to the improvement and consolidation of health surveillance management, especially in the planning, monitoring and evaluation of surveillance actions, in a timely manner.

For the purposes of violence surveillance, the *linkage* with different HIS contributes to the production of more robust information, a better understanding of the trajectories of women in health services, the identification of the determinants of violence and the worsening of cases, including the identification of possible risk profiles for femicide. This procedure also makes it possible to analyze the work processes developed in the services related to the care and referral of cases, promoting the identification of obstacles and missed opportunities for the health sector in dealing with violence.

The linking of databases has been considered strategic for the development of public policies in Brazil. Some authors suggest that this type of linking be done periodically, including various health databases, as well as those from other areas, such as public security, justice and social assistance^(41,43,46)^. However, when linking databases, it is imperative to consider the provisions of the General Data Protection Law (LGPD in the Portuguese acronym), which establishes strict guidelines for the processing and sharing of personal data, with the aim of protecting the privacy and security of citizens’ information. In addition, *linkage* is not a simple activity, requiring qualified staff, access to data and equipment to process large databases.

## PROSPECTS FOR THE FUTURE

In addition to the lack of interoperability between the HIS, analyses of the current context of SINASC, SINAN and SIM point to important limitations, such as the lack of standardization between fields, the existence of *offline* versions, obsolete language and low capacity to adapt to changes^(47)^.

In order to overcome these problems, the Ministry of Health launched the e-SUS Lifeline Program, which is scheduled to be implemented in 2025. The expectation is that this initiative will be the single, reliable source of birth, notification and death data^(35)^.

This proposal represents a change in the focus of the HIS, by focusing on the individual rather than the diseases or other health problems^(35)^, which will bring benefits to the field of violence studies and surveillance. It is hoped that integrated data will make it possible to deepen our understanding of the impact of violence on health, increase the detection of this event, take timely action and contribute to surveillance actions and scientific research^(35)^.

The Program’s main strengths are: data standardization, based on the definition of a minimum set of data (CMD in the Portuguese acronym); the adoption of standardized terminology; the issuing of electronic declarations; the immediate sending of typed data; timely updates; and the production of automated reports^(35,47)^.

However, it is crucial to recognize that these systems are not only essential tools for managing public policies; they are also products of continuous interactions between advances in information technology and the organizational and political context. Therefore, it is hoped that the implementation of the program will gain relevance on the public agenda and continuous investment to ensure its realization.

## FINAL CONSIDERATIONS

The five national HISs discussed in this article allow cases of violence to be recorded explicitly, such as notifications of interpersonal or self-inflicted violence in the SINAN; deaths due to self-inflicted, interpersonal or undetermined intent aggression in the SIM; primary and secondary diagnoses in hospitalizations and outpatient procedures in the SIH and SIA; and cases of rape of the vulnerable in girls under 14 with live-born children in the SINASC.

However, the complexity of violence against women requires an integrated approach to data, which has been achieved through *linkages* between the different HIS. Although this strategy is currently possible, there are limitations to its results, due to the completeness and quality of the original data.

The launch of the e-SUS Lifeline Program by the Ministry of Health represents an important step forward in the quest to integrate health information systems. By focusing on the individual and not just the disease, the program aims to overcome the problems of fragmentation and lack of standardization, becoming a single, reliable source of birth, notification and death data. This is a paradigm shift in health surveillance, in which the centrality is now the individual, a change that follows the not recent trend in the model of attention and care set out in public health policies in Brazil. It is therefore hoped that this perspective of health surveillance can add to the need to offer women in situations of violence comprehensive and humanized care, linking the detection, care and appropriate referral of cases and ensuring a sensitive approach to their specificities.

On the other hand, it is important to point out that the existing records alone, as highlighted at the beginning of this section, would already enable more effective action to be taken in dealing with cases of violence against women in the health sector, for example, by producing information and returning it to primary and/or specialized care. Or the follow-up of cases using systems that record procedures, for example in the case of sexual violence.

It is therefore to be hoped that the changes underway, which are important and desirable but may take time, do not block the potential that the systems have, even individually, to provide valuable data and information for tackling violence against women, a phenomenon which urgently requires more effective action from the Brazilian state.

## Footnotes

1 In Brazil, femicide gained prominence in the public policy sphere when Law 13.104 came into force in 2015, classifying it as a homicide committed against a woman because of her status as a female, i.e. when it involves domestic and family violence or contempt for or discrimination against women.
