# Supplementary material for: Health information systems and tackling violence against women: a necessary dialogue
Source: Rev Esc Enferm USP. 2025 Jun 13;59:e20240353. doi: 10.1590/1980-220X-REEUSP-2024-0353en (PMC12176325; doi:10.1590/1980-220X-REEUSP-2024-0353en)
Supplement: Translation

# Translation: Sistemas de informação em saúde e o enfrentamento das violências contra as mulheres: um diálogo necessário

**DOI:** 10.1590/1980-220X-REEUSP-2024-0353pt

**Authors:** Alice Cristina Medeiros Melo, Isabella Vitral Pinto, Camila Alves Bahia, Carmen Sant Fruchtman, Taynãna César Simões, Daniel Cobos, Letícia de Oliveira Cardoso, Paula Dias Bevilacqua

**Affiliations:** 1 Ministério da Saúde, Secretaria de Vigilância em Saúde e Ambiente, Departamento de Análise Epidemiológica e Vigilância de Doenças Não Transmissíveis, Brasília, DF, Brasil.; 2 Fundação Oswaldo Cruz, Instituto René Rachou, Grupo de Pesquisa Violências, Gênero e Saúde. Belo Horizonte, MG, Brasil.; 3 Universität Basel, Schweizerisches Tropen- und Public Health-Institut, Allschwil, Schweiz.; 4 Fundação Oswaldo Cruz, Instituto René Rachou, Núcleo de Estudos em Saúde Pública e Envelhecimento. Belo Horizonte, MG, Brasil.

**Keywords:** Banco de Dados, Vigilância em Saúde Pública, Direitos da Mulher, Assistência Integral à Saúde.

## Abstract

**Objetivo:**

Identificar informações obtidas de Sistemas de Informação em Saúde (SIS) que possibilitem detectar violências contra mulheres, discutindo criticamente estratégias para promover a integração desses dados e o aprimoramento das análises sobre violências.

**Método:**

Ensaio teórico reflexivo, utilizando revisão narrativa, pautado na análise de documentos nacionais sobre cinco SIS e suas potencialidades na detecção de violências contra as mulheres. Discutiram-se estratégias desenvolvidas no âmbito da gestão em saúde e na academia para promover a integração e disponibilização de dados e informações sobre a violência contra as mulheres.

**Resultados:**

Os cinco SIS descritos (SIM, SINAN, SINASC, SIH e SIA) apresentam possibilidades na construção de diagnósticos e no enfrentamento da violência contra mulheres que podem ser mais bem exploradas pela vigilância em saúde.

**Considerações finais:**

A integração de banco de dados para produção de informações é estratégica para o enfrentamento da violência contra as mulheres, permitindo o acompanhamento dos casos e a oferta de atendimento mais integral e humanizado às mulheres, colaborando para a prevenção de eventos extremos como o feminicídio.

## INTRODUÇÃO

Os Sistemas de Informação em Saúde (SIS) são ferramentas indispensáveis para a tomada de decisão, o planejamento, o monitoramento e a avaliação da situação e das ações em saúde^(1)^. No Brasil, o desenvolvimento dos SIS de abrangência nacional acompanhou o avanço das políticas públicas de saúde e as necessidades específicas de diferentes áreas no Ministério da Saúde (MS), bem como a evolução da tecnologia da informação^(2,3)^.

Os SIS em operação no Brasil desempenham um papel crucial ao fornecer informações clínicas e de atendimento, bem como dados essenciais para a formulação e o monitoramento de indicadores de morbimortalidade. Esses sistemas são fontes valiosas de evidências para análises da situação de saúde, sendo fundamentais para a condução de estudos que visam caracterizar e analisar epidemiologicamente doenças e agravos^(4)^.

O uso dos SIS para análise de situação das violências ganhou visibilidade em 2001, a partir da publicação da Política Nacional de Redução da Morbimortalidade por Acidentes e Violências^(5)^, e, em 2006, a partir da constituição do Sistema de Vigilância de Violências e Acidentes (VIVA). Esses marcos se somaram a outras ações promovidas pelo governo brasileiro em resposta à necessidade de desenvolvimento de políticas para o enfrentamento da violência no âmbito do setor saúde, destacando o papel dos SIS para o monitoramento e prevenção desse agravo^(6)^. Especificamente no campo da violência de gênero, a Política Nacional de Enfrentamento da Violência contra as Mulheres^(7)^ considera como ações prioritárias a produção e a sistematização de dados e informações sobre as violências, sendo essas atividades realizadas por múltiplos setores como saúde, segurança pública, justiça e assistência social.

Um estudo baseado em análise documental identificou a existência de 54 diferentes SIS de base nacional em funcionamento, entre 2010 e 2018, sob a gestão do Ministério da Saúde^(2)^, além de sistemas de iniciativas estaduais e municipais desenhados para atender especificidades e necessidades locais. Meninas e mulheres acessam os serviços de saúde para cuidados de rotina ou de emergência. Como a violência provoca consequências na saúde física, mental, reprodutiva e, ou, sexual, podendo também afetar o manejo de doenças crônicas^(8)^, espera-se que as mulheres em situação de violência procurem mais os serviços de saúde^(9)^. Assim, é fundamental que esses serviços tenham capacidade de detectar os casos e registrá-los nos sistemas de informação disponíveis em cada nível de atenção. Contudo, é importante destacar que apesar da variedade de sistemas de informação disponíveis, as informações sobre as mulheres e os eventos relacionados à violência frequentemente se apresentam de forma fragmentada nos sistemas^(10)^. Também, os sistemas têm operacionalidades independentes, não havendo compartilhamento de dados e informações entre eles^(11,12)^. Essa fragmentação limita a compreensão abrangente e longitudinal do fenômeno, dificultando os processos de tomada de decisão e ação no âmbito da vigilância e do cuidado em saúde.

A literatura científica brasileira é rica em artigos que analisam as características das violências e dos indivíduos em situação de violência a partir, principalmente, dos dados de notificação e de óbito^(13)^. Entretanto, é escassa a produção de estudos que discutem as contribuições dos SIS para o monitoramento das violências contra as mulheres a partir de uma abordagem integrada e longitudinal dos sistemas, capacidade para dar resposta. Dentre os estudos identificados, mencionamos o de Okabe e Fonseca^(10)^ e o Carvalho et al.^(14)^, o primeiro faz análises individualizadas do Sistema de Informação de Agravos de Notificação (SINAN), Sistema de Informações Hospitalares (SIH) e Sistema de Informação sobre Mortalidade (SIM), ou seja, discute as contribuições de cada um dos sistemas para o monitoramento e a tomada de decisão para o enfrentamento da violência. O segundo, é uma revisão integrativa utilizando de 25 estudos realizados no Brasil e em outros países, sendo que os estudos selecionados no Brasil analisam especialmente dados do SINAN. Embora tragam contribuições, esses estudos não abordam os desafios e as estratégias para a integração dos dados. Além disso, faz-se necessária a constante atualização sobre o tema, já que o enfrentamento da violência contra as mulheres continua sendo um grande desafio para a saúde e outros setores, seja pela magnitude, gravidade e, ou, agilidade de produzir novas formas de violência.

Assim, neste artigo, a partir de cinco SIS de base nacional (Sistema de Informações sobre Nascidos Vivos - SINASC; Sistema de Informação de Agravos de Notificação – SINAN; Sistema de Informação Hospitalar – SIH; Sistema de Informações Ambulatoriais – SIA; e Sistema de Informações sobre Mortalidade - SIM), atualizamos a discussão sobre as informações que esses SIS podem fornecer sobre as violências em geral e, quando pertinente, sobre tipos específicos de violência praticadas contra mulheres. Adicionalmente, discutimos criticamente estratégias que têm sido adotadas para promover a integração desses dados e o aprimoramento das análises sobre violências contra as mulheres por meio do uso integrado de SIS. Considerando a importância desse fenômeno, que apresenta magnitudes diversas e que produz diferentes impactos na saúde, bem como implicações políticas, econômicas, sociais, e culturais, seja para as mulheres sobreviventes ou para os casos de violência extrema, como o feminicídio, é importante contar com sistemas de informação que permitam a melhor compreensão dos casos de violência e eventos consequentes, como o óbito.

Como abordagem metodológica, realizamos uma revisão narrativa de literatura, a partir da seleção de artigos científicos publicados em diferentes periódicos e documentos técnico-científicos elaborados pelo Ministério da Saúde. A revisão narrativa de literatura é um método que utiliza fontes de informação diversas, mas não propõe esgotar a busca por trabalhos que tratam do assunto objeto da revisão, já que não utiliza procedimentos controlados de busca de dados^(15)^. A opção por publicações nacionais neste estudo se justifica pelo fato de nosso interesse ser refletir sobre a realidade brasileira em se tratando da capacidade de produzir informações e informar políticas públicas a partir dos sistemas de informação da saúde existentes.

## OPORTUNIDADES DE MONITORAR A VIOLÊNCIA CONTRA AS MULHERES NOS SISTEMAS DE INFORMAÇÃO EM SAÚDE

Tendo como referência a cronologia dos acontecimentos na vida, todos os nascimentos são registrados na Declaração de Nascido Vivo (DNV), documento-base do Sistema de Informações sobre Nascidos Vivos (SINASC). Esse sistema foi criado em 1990, com o intuito de superar os problemas de cobertura de registros de nascidos vivos, anteriormente feitos em cartórios de registro civil, padronizar a coleta dos dados em território nacional, além de ampliar os dados para permitir a construção e o acompanhamento de indicadores da situação de saúde materno-infantil^(16)^ (Quadro 1). No SINASC, é possível obter informações demográficas e epidemiológicas sobre o recém-nascido, a parturiente, o pré-natal e o parto^(17,18)^, servindo de base para o cálculo de indicadores como taxa de fecundidade, taxa de natalidade, taxa de mortalidade infantil e razão de morte materna^(19)^ (Quadro 1).

**Quadro 1:** Características dos Sistemas de Informação em Saúde e interfaces com a violência contra as mulheres – Belo Horizonte, MG, Brasil, 2024.

CARACTERÍSTICA/SIS	SINASC	SIA	SIH	SIM	SINAN
Ano de criação	1990	década de 1990		1975	2006 (evento violência)
Cronologia dos acontecimentos monitorados	Nascimento	Ao longo da vida	Ao longo da vida	Óbito	Ao longo da vida
Instrumento de registro	Declaração de Nascido Vivo (DN)	Boletim de Produção Ambulatorial Consolidado (BPA-C); Boletim de Produção Ambulatorial Individualizado (BPA-I); Autorização de Procedimentos Ambulatoriais (APAC)	–*	Declaração de Óbito (DO)	Ficha de notificação individual de violência interpessoal ou autoprovocadas
Objetivo	Registro de dados demográficas e epidemiológicas sobre o recém-nascido, a parturiente, o prénatal e o parto	Controle e o monitoramento dos atendimentos e dos tratamentos ambulatoriais	Registro e pagamento de internações hospitalares na rede pública e conveniada ao SUS	Registro de dados socioeconômicos do indivíduo, características do óbito e do profissional informante	Registro dos casos de violência para vigilância contínua
Cobertura	98,2%	–*	70% a 80% do total das internações	96,7%	–*
Tipo de dado	Individual	Agregado e individualizado	Individual	Individual	Individual
Possibilidade para monitorar a violência contra as mulheres	Gravidezes de meninas menores de 14 anos	Registro de dados específicos sobre violência sexual (a partir de 2014). Diagnósticos principal e secundário com base na CID-10. Procedimentos realizados que podem ser associados à violência, por exemplo, lesões, traumas, depressão, ansiedade, agravamento de doenças crônicas.	Análise conjunta dos diagnósticos principal e secundário do evento que levou à internação. Diagnóstico principal: dados sobre os tipos de lesões ou traumatismos, segundo capítulo XIX da CID-10. Diagnóstico secundário: caracterização do evento, segundo códigos do Capítulo XX da CID 10, por exemplo, se o evento é decorrente de acidente, violência interpessoal ou autoprovocada.	Monitoramento de feminicídios a partir das causas classificadas como agressões ou homicídios (códigos X85 a Y09 da CID-10)	Registro direto dos casos de violência. Registro de qualquer caso suspeito ou confirmado de violência doméstica ou intrafamiliar, sexual, autoprovocada, tráfico de pessoas, trabalho escravo e infantil, tortura, intervenção legal e violências homofóbicas contra mulheres e homens em todas as idades. Registro de casos de violência extrafamiliar ou comunitária contra crianças, adolescentes, mulheres, pessoas idosas, pessoas com deficiência, indígenas e população LGBT.

Notas: *Dados não disponíveis.

Apesar desse sistema não possuir campo específico para registro ou relato de violência, a informação sobre a idade das parturientes pode revelar situação de estupro de vulnerável (Quadro 1). Conforme o Código Penal, esse crime é definido como a existência de conjunção carnal ou prática de outro ato libidinoso com menor de 14 anos, sendo “irrelevante eventual consentimento da vítima para a prática do ato, sua experiência sexual anterior ou existência de relacionamento amoroso com o agente”^(20)^. Considerando apenas o registro do SINASC, a presunção (ou detecção) da violência sexual, seguida de gestação decorrente da violência, só ocorre no momento do parto.

Considerando a situação de vulnerabilidade de meninas e adolescentes, bem como a presunção de violência sexual, os dados da Declaração de Nascido Vivo (DNV) oferecem a possibilidade de identificar diversos aspectos, como escolaridade, raça/cor da pele, estado civil, histórico de gestações anteriores, qualidade do pré-natal (incluindo o momento em que começou e o número de consultas realizadas), bem como informações relacionadas ao parto e ao recém-nascido (como peso ao nascer e índice de Apgar). Além da importância desses dados para a produção de uma caracterização epidemiológica abrangente, também são cruciais para orientar a atuação da atenção primária à saúde, oferecendo informações para a organização do cuidado integral para as mães e seus bebês. Estudo publicado em 2024 utilizou dados do SINASC para analisar a distribuição espacial da gravidez em meninas menores de 14 anos de idade, revelando a maior vulnerabilidade com relação à violência sexual em residentes das regiões Norte e Centro-Oeste do Brasil^(21)^.

Em 2020, a cobertura do SINASC, no Brasil, foi de 98,2%, entretanto há grande heterogeneidade entre os municípios, especialmente nas Regiões Norte e Nordeste, que apresentaram os menores percentuais de cobertura, 95,5% e 96,5%, respectivamente^(19)^ (Quadro 1).

O registro das morbidades que ocorrem ao longo da vida do indivíduo é realizado em diferentes sistemas, como Sistema de Informações Ambulatoriais (SIA), Sistema de Informações Hospitalares (SIH) e Sistema de Informação de Agravos de Notificação (SINAN).

O SIA foi implementado na década de 1990, visando ao controle e ao monitoramento dos atendimentos e dos tratamentos ambulatoriais, por meio do Sistema de Gerenciamento da Tabela de Procedimentos do Sistema Único de Saúde (SIGTAP/SUS). Esse sistema registra desde consultas de atendimento básico até procedimentos realizados por prontos-socorros e ambulatórios de urgência/emergência, para o faturamento dos serviços de saúde realizados no SUS, definindo, assim, o repasse de recursos financeiros entre União, estados e municípios^(22)^ (Quadro 1).

A partir do SIA podemos acessar informações de forma agregada (Boletim de Produção Ambulatorial Consolidado/BPA-C) e individualizada (Boletim de Produção Ambulatorial Individualizado/BPA-I e Autorização de Procedimentos Ambulatoriais/APAC)^(22)^. Em 2014, foi criado o procedimento “atendimento multiprofissional para atenção integral às pessoas em situação de violência sexual” na tabela SIGTAP, que deve ser registrado no BPA-I e inclui agressão sexual por meio de força física; abuso sexual; problemas relacionados com abuso sexual alegado por uma criança e praticado por pessoa dentro de seu grupo familiar; e problemas relacionados com abuso sexual alegado por uma criança e praticado por pessoa de fora de seu grupo familiar^(23)^ (Quadro 1).

Além desse procedimento, que informa especificamente a situação de violência sexual, os diagnósticos principal e secundário, registrados com base na Classificação Internacional de Doenças - 10ª Edição (CID-10), bem como procedimentos realizados conforme a demanda do atendimento, podem ser relacionados a efeitos (agudos e crônicos) da violência (Quadro 1). Tais efeitos incluem lesões, traumas, depressão, ansiedade, agravamento de doenças crônicas, conforme demonstram diferentes estudos^(8,24)^.

Entretanto, as autoras e autores deste estudo desconhecem trabalhos que explorem os dados do SIA para avaliar procedimentos relacionados às violências contra as mulheres. Nas 25 produções científicas selecionadas em uma revisão integrativa, não houve menção ou análise de dados ambulatoriais para caracterização de casos de violências contra as mulheres^(14)^.

Nos eventos de saúde que requerem internações hospitalares, o SIH é o sistema de referência, sendo utilizado para o registro e o pagamento dessas ocorrências na rede pública (federal, estadual, municipal) e conveniada ao SUS^(25)^, apresentando cobertura de cerca de 70% a 80% do total das internações no país^(26)^ (Quadro 1).

A partir do SIH, podemos obter informações sobre o diagnóstico principal e secundário com base na CID-10, além de informações demográficas e geográficas para cada internação, apoiando o planejamento das ações de saúde e a produção de dados epidemiológicos (morbidade/mortalidade hospitalar)^(25)^. O diagnóstico principal se refere ao principal motivo que levou à hospitalização; já o secundário se refere a todas as condições que se desenvolveram durante a hospitalização ou que afetaram a atenção recebida e/ou o tempo de permanência no hospital^(27)^ (Quadro 1).

Os casos de violência nos registros de internação de mulheres podem ser identificados a partir do diagnóstico principal, o qual informa os tipos de lesões ou traumatismos, segundo capítulo XIX da CID-10^(28)^. Contudo, a caracterização do evento se dá a partir do diagnóstico secundário, preenchido com os códigos do Capítulo XX da CID 10^(28)^, os quais informam, por exemplo, se o evento é decorrente de acidente, violência interpessoal ou autoprovocada, sendo imprescindível a análise conjunta de ambos os diagnósticos para identificação dos casos de violência.

Apesar da importância dos dados hospitalares para caracterização e monitoramento dos eventos de violência^(5,10)^ e dos esforços empregados para qualificação do registro dos diagnósticos primário e secundário das internações^(10)^, pode-se afirmar que a produção científica com dados do SIH ainda é tímida em relação ao que é produzido com dados de mortalidade e de notificação.

O óbito, como último evento na vida de uma pessoa, é registrado no Sistema de Informação sobre Mortalidade (SIM) por meio da Declaração de Óbito (DO). O SIM, criado em 1975, é o sistema mais consolidado no Brasil, apresentando, em 2020, taxas de cobertura de 96,7%^(16)^ (Quadro 1). Entretanto, as taxas de cobertura entres as regiões brasileiras apresentam heterogeneidades importantes: Sul (100%), Sudeste (98,4%), Centro-Oeste (96,7%), Nordeste (93,4%) e Norte (92,1%)^(19)^.

A DO é constituída por campos que informam dados socioeconômicos do indivíduo, características do óbito e do profissional informante. A partir das informações contidas na DO, é possível o cálculo de indicadores como razão de mortalidade materna, taxas de mortalidade geral e específicas (segundo grupos etários, sexo, causas do óbito) e proporção de mortalidade por causa^(19)^. Esse sistema permite a identificação da violência extrema contra as mulheres, ou seja, a violência que resulta em um evento fatal (Quadro 1).

O preenchimento da DO é de exclusividade dos médicos^(29)^, sendo que, nos casos de óbito devido a causa externa, a emissão da DO é de competência dos médicos dos Institutos Médico-Legais, serviços vinculados à Segurança Pública nos estados.

Para a análise de óbitos decorrentes de violência contra mulheres, ou seja, feminicídio^1^, em geral, são selecionadas as causas classificadas como agressões ou homicídios (códigos X85 a Y09 da CID-10)^(30)^.

Entretanto, na DO, os feminicídios consumados não podem ser diferenciados dos homicídios, uma vez que não existe classificação específica na CID-10 para essa causa de morte. Nesses casos, já que o feminicídio é um qualificador do homicídio, esses óbitos acabam sendo registrados com os códigos X85 a Y09. Alguns estudos vêm investindo esforços para a visibilização do feminicídio a partir dos dados do SIM, a exemplo do Atlas da Violência, que, desde a edição 2020, considera o evento ‘homicídio de mulheres nas residências’ como medida indireta dos casos de feminicídio^(31)^.

Ainda, óbitos de mulheres classificados como suicídio, acidentes e mesmo causas mal definidas e de intenção indeterminada, podem ocultar situações de violência interpessoal que levaram ao óbito. Por isso, a investigação do óbito de mulheres é importante, requerendo análises específicas desses registros, ou, ainda, relacionando-os com dados da segurança pública para a adequada qualificação do óbito.

Considerando a violência contra as mulheres, o Sistema de Informação de Agravos de Notificação (SINAN) é aquele em que esse evento é registrado de forma direta e sistemática, por meio da Ficha de Notificação Individual. A vigilância da violência foi implementada pelo MS, em 2006, a partir do Sistema de Vigilância de Violências e Acidentes (VIVA), constituído por dois componentes: a vigilância sentinela e a vigilância contínua^(32)^. A vigilância contínua se tornou compulsória a partir de 2011, por meio da inserção da violência no rol de agravos de notificação compulsória^(33)^ devendo ser registrada no SINAN por profissionais de saúde de serviços públicos e privados. A notificação deve ser realizada para qualquer caso suspeito ou confirmado de violência doméstica/intrafamiliar, sexual, autoprovocada, tráfico de pessoas, trabalho escravo e infantil, tortura, intervenção legal e violências homofóbicas contra mulheres e homens em todas as idades. No caso de violência extrafamiliar/comunitária, somente serão objetos de notificação as violências contra crianças, adolescentes, mulheres, pessoas idosas, pessoas com deficiência, indígenas e população LGBT^(32)^ (Quadro 1).

O SINAN supriu uma lacuna no registro do evento ‘violência’ pelo setor saúde, já que o caso é registrado de forma direta e não como consequência de um acontecimento, como atendimento, hospitalização ou óbito.

Desde a incorporação das violências como agravo de notificação compulsória, os dados do SINAN têm sido amplamente utilizados para análise e caracterização de indivíduos e dos diferentes eventos relacionados às violências contra as mulheres. Em que pese o esforço de consolidação e ampliação das notificações em todo território nacional^(6)^, a subnotificação ainda é um desafio para os serviços de saúde em todas as unidades federativas do Brasil^(34)^.

Considerando as características dos SIS apresentadas, percebe-se que esses sistemas foram concebidos e organizados de forma a atender demandas específicas, como, por exemplo, o registro de um evento/agravo ou procedimento e formas de funcionamento próprias do SUS. Apesar das limitações em termos de cobertura, completitude, qualidade dos dados, bem como a falta de interoperabilidade entre os sistemas, seus dados têm sido continuamente utilizados para a produção de diagnósticos relacionados às violências contra as mulheres. Nesse sentido, eles têm desempenhado um papel fundamental na visibilidade do problema e no planejamento de políticas, visando aprimorar as ações de enfrentamento da violência.

## O PAPEL DO *LINKAGE NA* BUSCA E DETECÇÃO DE VIOLÊNCIA CONTRA AS MULHERES

Dado que os SIS são projetados e estruturados com propósitos específicos, seja para a vigilância, a atenção e a gestão em saúde, muitas vezes de forma independente, podem ser encontradas redundância, discrepância ou complementaridade nos dados entre diferentes sistemas. Se por um lado essa diversidade de informações é benéfica, por outro, exige-se um investimento em análises integradas envolvendo os diversos bancos de dados.

Devido à ausência de um identificador único e à falta de interoperabilidade entre os SIS de base nacional^(35)^ abordados neste artigo, as áreas da pesquisa e da gestão têm recorrido ao relacionamento das bases de dados (*record linkage*) como estratégia para integrar as informações e obter uma visão mais abrangente dos casos de violência contra as mulheres. O uso de técnicas de relacionamento de registros pode ajudar a identificar padrões, compreender de forma mais aprofundada a dinâmica das violências e fornecer evidências importantes para o desenvolvimento de políticas e intervenções eficazes.

O *linkage* pode ser definido como um método de busca de pares idênticos em uma mesma base ou em bases de dados distintas^(36,37)^. A partir dessa técnica é possível encontrar pares de registros que se referem a uma mesma pessoa, bem como melhorar a completitude e a consistência dos dados^(36,37)^.

A ausência de um identificador único e consistente, que possibilite a localização do mesmo indivíduo em diferentes bancos de dados^(4)^, como o Cadastro de Pessoa Física (CPF), por exemplo, implica em maior complexidade, exigindo a utilização de duas ou mais variáveis-chaves, como nome e data de nascimento. Entretanto, nesses campos pode haver erros de digitação, uso de abreviações, valores faltantes, o que afetará a qualidade do resultado final do *linkage* ^(35,38)^. No caso da abordagem determinística, em que a concordância precisa ser total para a formação dos pares, pequenas diferenças nos nomes ou na data de nascimento resultariam no não encontro dos registros e em uma consequente perda de pares verdadeiros^(37)^. Já, na abordagem probabilística, é preciso determinar um ponto de corte para a avaliação da similaridade dos pares, sempre buscando a redução de falsos positivos e de falsos negativos.

Diversos estudos têm utilizado as técnicas de *linkage* para compreender as repercussões das violências na vida das mulheres, ampliar a detecção dos casos, bem como avaliar riscos de desfechos evitáveis. Nos Estados Unidos, Cerel et al.^(39)^ utilizaram o *linkage* para analisar a relação entre mortes violentas e admissões anteriores nos serviços de emergência, por lesões decorrentes de causas externas. A técnica também foi utilizada para localizar os óbitos maternos que estavam associados às mortes violentas na Carolina do Norte/EUA^(40)^. No Brasil, já se aplicou o *linkage* para analisar os óbitos por agressão^(41)^, a violência sexual e suas posteriores repercussões na gravidez e parto^(42)^, mulheres com notificação de violência por parceiro íntimo e óbito por qualquer causa básica^(43)^, risco de óbito em mulheres com notificação de violência interpessoal^(44)^ e mortalidade de mulheres com notificação de violência durante a gravidez^(45)^.

A integração dos sistemas de informações em saúde é inclusive considerada uma importante estratégia da Política Nacional de Vigilância em Saúde (PNVS), no sentido de contribuir para o aprimoramento e a consolidação da gestão da vigilância em saúde, especialmente nas atividades de planejamento, monitoramento e avaliação das ações de vigilância, em tempo oportuno.

Para a vigilância das violências, o *linkage* com diferentes SIS contribui para a produção de informações mais robustas, melhor compreensão das trajetórias das mulheres nos serviços de saúde, identificação dos determinantes das violências e do agravamento dos casos, inclusive identificando possíveis perfis de risco para o feminicídio. Esse procedimento também permite analisar os processos de trabalho desenvolvidos nos serviços relacionados ao atendimento e ao encaminhamento dos casos, promovendo a identificação de obstáculos e perdas de oportunidades do setor saúde na abordagem da violência.

O relacionamento das bases de dados tem sido considerado estratégico para a elaboração de políticas públicas no Brasil. Alguns autores sugerem que esse tipo de relacionamento seja feito periodicamente, incluindo diversas bases de dados da saúde, bem como de outras áreas, como da segurança pública, justiça e assistência social^(41,43,46)^. No entanto, ao realizar o relacionamento entre as bases de dados, é imperativo considerar as disposições da Lei Geral de Proteção de Dados (LGPD), que estabelece diretrizes rigorosas para o tratamento e compartilhamento de dados pessoais, visando proteger a privacidade e a segurança das informações dos cidadãos. Além disso, a realização do *linkage* não é uma atividade simples, exigindo equipe qualificada, acesso aos dados e equipamentos que permitam o tratamento de grandes bancos de dados.

## PERSPECTIVAS PARA O FUTURO

Para além da falta de interoperabilidade entre os SIS, as análises do contexto atual do SINASC, SINAN e SIM apontam para importantes limitações, como a falta de padronização entre campos, existência de versões *off-line*, linguagem obsoleta e baixa capacidade de adaptação a mudanças^(47)^.

Com intuito de superar esses problemas, o Ministério da Saúde lançou o Programa e-SUS Linha da Vida, cuja implementação está prevista para 2025. A expectativa é que essa iniciativa seja a fonte única e confiável dos dados de nascimento, notificações e óbito^(35)^.

Essa proposta representa uma mudança do enfoque dos SIS, ao direcionar o olhar para o indivíduo e não para a doença/agravo^(35)^, o que trará benefícios para o campo dos estudos e vigilância de violências. Espera-se que com os dados integrados seja possível aprofundar a compreensão dos impactos das violências na saúde, ampliar a detecção desse evento, atuar em tempo oportuno e contribuir para as ações de vigilância e a realização de pesquisas científicas^(35)^.

Destacam-se como as principais potencialidades do Programa: a padronização dos dados, a partir da definição de um conjunto mínimo de dados (CMD); a adoção de terminologias padronizadas; a emissão de declarações eletrônicas; o envio imediato dos dados digitados; a atualizações realizadas em tempo oportuno; e a produção de relatórios automatizados^(35,47)^.

No entanto, é crucial reconhecer que esses sistemas não são apenas ferramentas essenciais para a gestão das políticas públicas; são também produtos de contínuas interações entre os avanços da tecnologia da informação e o contexto organizacional e político. Portanto, espera-se que a implementação do programa ganhe relevância na agenda pública e investimentos contínuos para garantir sua concretização.

## CONSIDERAÇÕES FINAIS

Os cinco SIS de base nacional abordados neste artigo permitem o registro de casos de violência de forma explícita, como as notificações de violência interpessoal ou autoprovocada no SINAN; os óbitos por agressão autoprovocada, interpessoal ou de intenção indeterminada no SIM; os diagnósticos, principal e secundário, nas internações e nos procedimentos ambulatoriais, no SIH e SIA; e os casos de estupro de vulnerável em meninas com menos de 14 anos com filhos nascidos vivos no SINASC.

Entretanto, a complexidade da violência contra as mulheres demanda uma abordagem integrada dos dados, o que tem sido alcançado por meio da realização de *linkages* entre os diferentes SIS. Apesar de ser a estratégia possível, atualmente, destaca-se que há limitações nos seus resultados, advindos da completitude e qualidade dos dados originais.

O lançamento do Programa e-SUS Linha da Vida pelo Ministério da Saúde representa um avanço importante na busca pela integração dos sistemas de informação em saúde. Ao direcionar o foco para o indivíduo e não apenas para a doença/agravo, o programa visa superar os problemas de fragmentação e falta de padronização, tornando-se uma fonte única e confiável de dados de nascimento, notificações e óbito. Esse direcionamento não deixa de ser uma mudança de paradigma na vigilância em saúde, em que a centralidade passa a ser o indivíduo, mudança que segue a tendência, não recente, do modelo de atenção e cuidado previsto nas políticas públicas de saúde no Brasil. Tem-se, assim, como expectativa, que essa perspectiva da vigilância em saúde possa somar esforços à necessidade de ser oferecido às mulheres em situação de violência um atendimento integral e humanizado, articulando a detecção, o atendimento e o encaminhamento adequados dos casos e garantindo um olhar sensível às suas especificidades.

Por outro lado, é importante ressaltar que apenas os registros já existentes, conforme destacado no início dessa seção, já permitiriam uma atuação mais efetiva na abordagem dos casos de violência contra as mulheres no setor saúde, por exemplo, a partir da produção de informações e retorno das mesmas, para a atenção primária e/ou especializada. Ou o acompanhamento dos casos utilizando os sistemas que registram os procedimentos, por exemplo, no caso da violência sexual.

Assim, espera-se que as mudanças em curso, importantes e desejáveis, porém, eventualmente, demoradas, não obliterem o potencial que os sistemas têm, mesmo que individualmente, de fornecer dados e informações preciosas para o enfrentamento das violências contra as mulheres, fenômeno para o qual são urgentes ações mais efetivas do estado brasileiro.

## Footnotes

1 No Brasil, o feminicídio ganhou destaque no âmbito das políticas públicas quando, em 2015, entrou em vigor a Lei nº 13.104, que o qualificou como o homicídio cometido contra a mulher por razões da condição de sexo feminino, ou seja, quando envolve violência doméstica e familiar ou menosprezo ou discriminação à condição de mulher.

# Translation: Sistemas de información en salud y enfrentamiento de la violencia contra las mujeres: un diálogo necesario

**DOI:** 10.1590/1980-220X-REEUSP-2024-0353es

**Authors:** Alice Cristina Medeiros Melo, Isabella Vitral Pinto, Camila Alves Bahia, Carmen Sant Fruchtman, Taynãna César Simões, Daniel Cobos, Letícia de Oliveira Cardoso, Paula Dias Bevilacqua

**Affiliations:** 1 Ministério da Saúde, Secretaria de Vigilância em Saúde e Ambiente, Departamento de Análise Epidemiológica e Vigilância de Doenças Não Transmissíveis, Brasília, DF, Brasil.; 2 Fundação Oswaldo Cruz, Instituto René Rachou, Grupo de Pesquisa Violências, Gênero e Saúde. Belo Horizonte, MG, Brasil.; 3 Universität Basel, Schweizerisches Tropen- und Public Health-Institut, Allschwil, Schweiz.; 4 Fundação Oswaldo Cruz, Instituto René Rachou, Núcleo de Estudos em Saúde Pública e Envelhecimento. Belo Horizonte, MG, Brasil.

**Keywords:** Base de Datos, Vigilancia en Salud Pública, Derechos de la Mujer, Asistencia Integral a la Salud.

## Abstract

**Objetivo:**

Identificar la información obtenida de los Sistemas de Información en Salud (SIS) que permita detectar la violencia contra las mujeres, discutiendo críticamente estrategias para promover la integración de estos datos y el perfeccionamiento de los análisis sobre la violencia.

**Método:**

Ensayo teórico reflexivo, utilizando una revisión narrativa, basado en el análisis de documentos nacionales sobre cinco SIS y sus potencialidades en la detección de la violencia contra las mujeres. Se discutieron estrategias desarrolladas en el ámbito de la gestión en salud y en el ámbito académico para promover la integración y disponibilidad de datos e información sobre la violencia contra las mujeres.

**Resultados:**

Los cinco SIS descritos (SIM, SINAN, SINASC, SIH y SIA) presentan posibilidades en la construcción de diagnósticos y en el enfrentamiento de la violencia contra las mujeres que pueden ser mejor exploradas por la vigilancia en salud.

**Consideraciones finales:**

La integración de bases de datos para la producción de información es estratégica para hacer frente a la violencia contra las mujeres, ya que permite el seguimiento de los casos y la oferta de una atención más integral y humanizada a las mujeres, colaborando en la prevención de eventos extremos como el feminicidio.

## INTRODUCCIÓN

Los Sistemas de Información en Salud (SIS) son herramientas indispensables para la toma de decisiones, la planificación, el seguimiento y la evaluación de la situación y las acciones en salud^(1)^. En Brasil, el desarrollo de los SIS de alcance nacional ha acompañado el avance de las políticas públicas de salud y las necesidades específicas de diferentes áreas del Ministerio de Salud (MS), así como la evolución de la tecnología de la información^(2,3)^.

Los SIS en funcionamiento en Brasil desempeñan un papel crucial al proporcionar información clínica y de atención, así como datos esenciales para la formulación y el seguimiento de indicadores de morbimortalidad. Estos sistemas son fuentes valiosas de evidencia para el análisis de la situación sanitaria y son fundamentales para la realización de estudios que tienen por objeto caracterizar y analizar epidemiológicamente enfermedades y afecciones^(4)^.

El uso de los SIS para el análisis de la situación de la violencia ganó visibilidad en 2001, a partir de la publicación de la Política Nacional de Reducción de la Morbimortalidad por Accidentes y Violencia^(5)^, y en 2006, a partir de la constitución del Sistema de Vigilancia de Violencia y Accidentes (VIVA). Estos hitos se sumaron a otras acciones promovidas por el Gobierno brasileño en respuesta a la necesidad de desarrollar políticas para hacer frente a la violencia en el ámbito del sector salud, destacando el papel de los SIS para el seguimiento y la prevención de este problema^(6)^. Específicamente en el ámbito de la violencia de género, la Política Nacional de Enfrentamiento a la Violencia contra las Mujeres^(7)^ considera como acciones prioritarias la producción y sistematización de datos e información sobre la violencia, actividades que se llevan a cabo en múltiples sectores, como salud, seguridad pública, justicia y asistencia social.

Un estudio basado en el análisis documental identificó la existencia de 54 SIS diferentes a nivel nacional en funcionamiento entre 2010 y 2018, bajo la gestión del Ministerio de Salud^(2)^, además de sistemas de iniciativas estatales y municipales diseñados para atender las especificidades y necesidades locales. Las niñas y las mujeres acceden a los servicios de salud para recibir atención rutinaria o de emergencia. Dado que la violencia tiene consecuencias en la salud física, mental, reproductiva y/o sexual, y también puede afectar al manejo de enfermedades crónicas^(8)^, se espera que las mujeres en situación de violencia acudan más a los servicios de salud^(9)^. Por lo tanto, es fundamental que estos servicios tengan la capacidad de detectar los casos y registrarlos en los sistemas de información disponibles en cada nivel de atención. Sin embargo, es importante destacar que, a pesar de la variedad de sistemas de información disponibles, la información sobre las mujeres y los eventos relacionados con la violencia a menudo se presenta de forma fragmentada en los sistemas^(10)^. Además, los sistemas tienen operatividades independientes, sin que exista un intercambio de datos e información entre ellos^(11,12)^. Esta fragmentación limita la comprensión global y longitudinal del fenómeno, dificultando los procesos de toma de decisiones y de acción en el ámbito de la vigilancia y la atención en salud.

La literatura científica brasileña es rica en artículos que analizan las características de la violencia y de las personas en situación de violencia a partir, principalmente, de los datos de notificación y de defunción^(13)^. Sin embargo, son escasos los estudios que discuten las contribuciones de los SIS para el monitoreo de la violencia contra las mujeres a partir de un enfoque integrado y longitudinal de los sistemas, capacidad para dar respuesta. Entre los estudios identificados, cabe mencionar el de Okabe y Fonseca^(10)^ y el Carvalho et al.^(14)^. El primero realiza análisis individualizados del Sistema de Información sobre Agraves de Notificación (SINAN), el Sistema de Información Hospitalaria (SIH) y el Sistema de Información sobre Mortalidad (SIM), es decir, discute las contribuciones de cada uno de los sistemas para el seguimiento y la toma de decisiones para hacer frente a la violencia. El segundo es una revisión integradora que utiliza 25 estudios realizados en Brasil y otros países, y los estudios seleccionados en Brasil analizan especialmente los datos del SINAN. Aunque aportan contribuciones, estos estudios no abordan los retos y las estrategias para la integración de los datos. Además, es necesario actualizar constantemente la información sobre el tema, ya que la lucha contra la violencia contra las mujeres sigue siendo un gran desafío para la salud y otros sectores, ya sea por su magnitud, gravedad o rapidez en la aparición de nuevas formas de violencia.

Así, en este artículo, a partir de cinco SIS de ámbito nacional (Sistema de Información sobre Nacidos Vivos - SINASC; Sistema de Información sobre Problemas de Salud de Notificación - SINAN; Sistema de Información Hospitalaria - SIH; Sistema de Información Ambulatoria - SIA; y Sistema de Información sobre Mortalidad - SIM), actualizamos el debate sobre la información que estos SIS pueden proporcionar sobre la violencia en general y, cuando procede, sobre tipos específicos de violencia ejercida contra las mujeres. Además, analizamos críticamente las estrategias que se han adoptado para promover la integración de estos datos y la mejora de los análisis sobre la violencia contra las mujeres mediante el uso integrado de los SIS. Teniendo en cuenta la importancia de este fenómeno, que presenta magnitudes diversas y produce diferentes impactos en la salud, así como implicaciones políticas, económicas, sociales y culturales, tanto para las mujeres sobrevivientes como para los casos de violencia extrema, como el feminicidio, es importante contar con sistemas de información que permitan comprender mejor los casos de violencia y los eventos consecuentes, como la muerte.

Como enfoque metodológico, realizamos una revisión narrativa de la literatura, a partir de la selección de artículos científicos publicados en diferentes periódicos y documentos técnico-científicos elaborados por el Ministerio de Salud. La revisión narrativa de la literatura es un método que utiliza diversas fuentes de información, pero no propone agotar la búsqueda de trabajos que traten el tema objeto de la revisión, ya que no utiliza procedimientos controlados de búsqueda de datos^(15)^. La elección de publicaciones nacionales en este estudio se justifica por el hecho de que nuestro interés es reflexionar sobre la realidad brasileña en lo que se refiere a la capacidad de producir información e informar las políticas públicas a partir de los sistemas de información de salud existentes.

## OPORTUNIDADES PARA EL MONITOREO DE LA VIOLENCIA CONTRA LAS MUJERES EN LOS SISTEMAS DE INFORMACIÓN EN SALUD

Tomando como referencia la cronología de los acontecimientos de la vida, todos los nacimientos se registran en la Declaración de Nacido Vivo (DNV), documento base del Sistema de Información sobre Nacidos Vivos (SINASC). Este sistema fue creado en 1990 con el objetivo de superar los problemas de cobertura de los registros de nacidos vivos, que anteriormente se realizaban en las oficinas del registro civil, estandarizar la recolección de datos en el territorio nacional y ampliar los datos para permitir la construcción y el seguimiento de indicadores de la situación de salud maternoinfantil^(16)^ (Cuadro 1). En el SINASC es posible obtener información demográfica y epidemiológica sobre el recién nacido, la parturienta, el prenatal y el parto^(17,18)^, que sirve de base para el cálculo de indicadores como la tasa de fecundidad, la tasa de natalidad, la tasa de mortalidad infantil y la razón de mortalidad materna^(19)^ (Cuadro 1).

**Cuadro 1:** Características de los Sistemas de Información en Salud e interfaces con la violencia contra la mujer – Belo Horizonte, MG, Brasil, 2024.

CARACTERÍSTICA/SISTEMA	SINASC	SIA	SIH	SÍ	SINAN
Año de creación	1990	1990s		1975	2006 (acto violento)
Cronología de los acontecimientos controlados	Nacimiento	A lo largo de la vida	A lo largo de la vida	Muerte	A lo largo de la vida
Instrumento de registro	Declaración de nacido vivo (DN)	Boletín consolidado de producción ambulatoria (BPA-C); Boletín individualizado de producción ambulatoria (BPA-I); Autorización de procedimientos ambulatorios (APAC)	–*	Certificado de defunción (DD)	Formulario de notificación individual de violencia interpersonal o autolesión
Objetivo	Registro de datos demográficos y epidemiológicos sobre el recién nacido, la parturienta, los cuidados prenatales y el parto.	Control y seguimiento de la atención y el tratamiento ambulatorios	Registro y pago de los ingresos hospitalarios en las redes públicas y afiliadas al SUS	Registro de datos socioeconómicos del individuo, características de la defunción y del profesional informador	Registro de casos de violencia para una vigilancia permanente
Portada	98,2%	–*	Entre el 70% y el 80% de todas las hospitalizaciones	96,7%	–*
Tipo de datos	Individual	Agregados e individualizados	Individual	Individual	Individual
Posibilidad de controlar la violencia contra las mujeres	Embarazos de niñas menores de 14 años	Registro de datos específicos sobre violencia sexual (a partir de 2014). Diagnósticos primarios y secundarios basados en la CIE-10. Procedimientos realizados que pueden asociarse a la violencia, por ejemplo, lesiones, traumatismos, depresión, ansiedad, agravamiento de enfermedades crónicas	Análisis conjunto de los diagnósticos primario y secundario del acontecimiento que dio lugar a la hospitalización. Diagnóstico principal: datos sobre los tipos de lesiones o traumatismos, según el capítulo XIX de la CIE-10. Diagnóstico secundario: caracterización del acontecimiento, según los códigos del capítulo XX de la CIE 10, por ejemplo, si el acontecimiento es consecuencia de un accidente, de violencia interpersonal o de autolesiones.	Seguimiento de los feminicidios por causas clasificadas como agresiones u homicidios (códigos CIE-10 X85 a Y09)	Registro directo de casos de violencia. Registro de todos los casos presuntos o confirmados de violencia doméstica o intrafamiliar, violencia sexual, violencia autoinfligida, trata de seres humanos, trabajo esclavo e infantil, tortura, intervención legal y violencia homófoba contra mujeres y hombres de todas las edades. Registro de casos de violencia extrafamiliar o comunitaria contra niños, adolescentes, mujeres, ancianos, personas con discapacidad, indígenas y población LGBT.

Notas: *Datos no disponibles.

Aunque este sistema no cuenta con un campo específico para registrar o denunciar la violencia, la información sobre la edad de las parturientas puede revelar situaciones de violación de personas vulnerables (Cuadro 1). Según el Código Penal, este delito se define como la existencia de relaciones carnales o la práctica de otro acto de naturaleza sexual con menores de 14 años, siendo «irrelevante el consentimiento eventual de la víctima para la práctica del acto, su experiencia sexual previa o la existencia de una relación amorosa con el agente»^(20)^. Teniendo en cuenta únicamente el registro del SINASC, la presunción (o detección) de violencia sexual, seguida de un embarazo derivado de la violencia, solo se produce en el momento del parto.

Teniendo en cuenta la situación de vulnerabilidad de las niñas y adolescentes, así como la presunción de violencia sexual, los datos de la Declaración de Nacidos Vivos (DNV) ofrecen la posibilidad de identificar diversos aspectos, como el nivel de escolaridad, la raza/color de la piel, el estado civil, los antecedentes de embarazos anteriores, la calidad de la atención prenatal (incluido el momento en que comenzó y el número de consultas realizadas), así como información relacionada con el parto y el recién nacido (como el peso al nacer y el índice de Apgar). Además de la importancia de estos datos para la elaboración de una caracterización epidemiológica exhaustiva, también son cruciales para orientar la actuación de la atención primaria de salud, ya que proporcionan información para la organización de la atención integral a las madres y sus bebés. Un estudio publicado en 2024 utilizó datos del SINASC para analizar la distribución espacial de los embarazos en niñas menores de 14 años, revelando una mayor vulnerabilidad con respecto a la violencia sexual en las residentes de las regiones Norte y Centro-Oeste de Brasil^(21)^.

En 2020, la cobertura del SINASC en Brasil fue del 98,2%, sin embargo, existe una gran heterogeneidad entre los municipios, especialmente en las regiones Norte y Nordeste, que presentaron los porcentajes más bajos de cobertura, 95,5% y 96,5%, respectivamente^(19)^ (Cuadro 1).

El registro de las morbilidades que se producen a lo largo de la vida del individuo se realiza en diferentes sistemas, como el Sistema de Información Ambulatoria (SIA), el Sistema de Información Hospitalaria (SIH) y el Sistema de Información de Problemas de Salud de Notificación (SINAN).

El SIA se implementó en la década de 1990 con el objetivo de controlar y supervisar la atención y los tratamientos ambulatorios a través del Sistema de Gestión de la Tabla de Procedimientos del Sistema Único de Salud (SIGTAP/SUS). Este sistema registra desde consultas de atención básica hasta procedimientos realizados en servicios de urgencias y ambulatorios de urgencia/emergencia, para la facturación de los servicios de salud prestados en el SUS, definiendo así la transferencia de recursos financieros entre la Unión, los estados y los municipios^(22)^ (Cuadro 1).

A partir del SIA podemos acceder a información de forma agregada (Boletín de Producción Ambulatoria Consolidada/BPA-C) e individualizada (Boletín de Producción Ambulatoria Individualizada/BPA-I y Autorización de Procedimientos Ambulatorios/APAC)^(22)^. En 2014 se creó el procedimiento «atención multiprofesional para la atención integral a personas en situación de violencia sexual» en la tabla SIGTAP, que debe registrarse en el BPA-I e incluye agresión sexual mediante fuerza física; abuso sexual; problemas relacionados con el abuso sexual denunciado por un niño y cometido por una persona de su grupo familiar; y problemas relacionados con el abuso sexual denunciado por un niño y cometido por una persona ajena a su grupo familiar^(23)^ (Cuadro 1).

Además de este procedimiento, que informa específicamente sobre la situación de violencia sexual, los diagnósticos principales y secundarios, registrados según la Clasificación Internacional de Enfermedades - 10.ª Edición (CIE-10), así como los procedimientos realizados según la demanda de la atención, pueden relacionarse con los efectos (agudos y crónicos) de la violencia (Cuadro 1). Dichos efectos incluyen lesiones, traumas, depresión, ansiedad y agravamiento de enfermedades crónicas, según demuestran diferentes estudios^(8,24)^.

Sin embargo, los autores de este estudio desconocen trabajos que exploren los datos del SIA para evaluar los procedimientos relacionados con la violencia contra las mujeres. En las 25 publicaciones científicas seleccionadas en una revisión integradora, no se mencionaron ni analizaron datos ambulatorios para caracterizar los casos de violencia contra las mujeres^(14)^.

En los eventos de salud que requieren hospitalización, el SIH es el sistema de referencia, utilizándose para el registro y el pago de estos eventos en la red pública (federal, estatal, municipal) y afiliada al SUS^(25)^, con una cobertura de alrededor del 70% al 80% del total de hospitalizaciones en el país^(26)^ (Cuadro 1).

A partir del SIH, podemos obtener información sobre el diagnóstico principal y secundario según la CIE-10, además de información demográfica y geográfica para cada hospitalización, lo que respalda la planificación de las acciones sanitarias y la producción de datos epidemiológicos (morbilidad/mortalidad hospitalaria)^(25)^. El diagnóstico principal se refiere al motivo principal que llevó a la hospitalización, mientras que el secundario se refiere a todas las afecciones que se desarrollaron durante la hospitalización o que afectaron a la atención recibida y/o al tiempo de permanencia en el hospital^(27)^ (Cuadro 1).

Los casos de violencia en los registros de hospitalización de mujeres pueden identificarse a partir del diagnóstico principal, que informa los tipos de lesiones o traumatismos, según el capítulo XIX de la CIE-10^(28)^. Sin embargo, la caracterización del evento se realiza a partir del diagnóstico secundario, completado con los códigos del capítulo XX de la CIE-10^(28)^, que informan, por ejemplo, si el evento es consecuencia de un accidente, violencia interpersonal o autoproducida, siendo imprescindible el análisis conjunto de ambos diagnósticos para identificar los casos de violencia.

A pesar de la importancia de los datos hospitalarios para la caracterización y el seguimiento de los eventos de violencia^(5,10)^ y de los esfuerzos realizados para mejorar el registro de los diagnósticos primarios y secundarios de las hospitalizaciones^(10)^, se puede afirmar que la producción científica con datos del SIH sigue siendo escasa en comparación con la que se produce con datos de mortalidad y notificación.

La muerte, como último evento en la vida de una persona, se registra en el Sistema de Información sobre Mortalidad (SIM) mediante la Declaración de Defunción (DD). El SIM, creado en 1975, es el sistema más consolidado en Brasil, con tasas de cobertura del 96,7%^(16)^ en 2020 (Cuadro 1). Sin embargo, las tasas de cobertura entre las regiones brasileñas presentan importantes heterogeneidades: Sur (100%), Sudeste (98,4%), Centro-Oeste (96,7%), Nordeste (93,4%) y Norte (92,1%)^(19)^.

El DD está constituido por campos que informan datos socioeconómicos del individuo, características de la muerte y del profesional informante. A partir de la información contenida en la DD, es posible calcular indicadores como la tasa de mortalidad materna, las tasas de mortalidad general y específica (según grupos de edad, sexo, causas de muerte) y la proporción de mortalidad por causa^(19)^. Este sistema permite identificar la violencia extrema contra las mujeres, es decir, la violencia que da lugar a un evento fatal (Cuadro 1).

El llenado de la DD es competencia exclusiva de los médicos^(29)^, y en los casos de muerte por causa externa, la emisión de la DD es competencia de los médicos de los Institutos Médico-Legales, servicios vinculados a la Seguridad Pública en los estados.

Para el análisis de las muertes derivadas de la violencia contra las mujeres, es decir, los feminicidios^1^, se seleccionan, en general, las causas clasificadas como agresiones u homicidios (códigos X85 a Y09 de la CIE-10)^(30)^.

Sin embargo, en la DD, los feminicidios consumados no pueden diferenciarse de los homicidios, ya que no existe una clasificación específica en la CIE-10 para esta causa de muerte. En estos casos, dado que el feminicidio es un calificador del homicidio, estas muertes terminan registrándose con los códigos X85 a Y09. Algunos estudios están invirtiendo esfuerzos para visibilizar el feminicidio a partir de los datos del SIM, como el Atlas de la Violencia, que, desde la edición de 2020, considera el evento «homicidio de mujeres en residencias» como una medida indirecta de los casos de feminicidio^(31)^.

Además, las muertes de mujeres clasificadas como suicidio, accidentes e incluso causas mal definidas y de intención indeterminada pueden ocultar situaciones de violencia interpersonal que condujeron a la muerte. Por ello, la investigación de la muerte de las mujeres es importante y requiere un análisis específico de estos registros o, incluso, su relación con datos de seguridad pública para la adecuada calificación de la muerte.

Teniendo en cuenta la violencia contra las mujeres, el Sistema de Información sobre Problemas de Salud Notificables (SINAN) es aquel en el que este evento se registra de forma directa y sistemática, mediante la Ficha de Notificación Individual. La vigilancia de la violencia fue implementada por el MS en 2006, a partir del Sistema de Vigilancia de Violencia y Accidentes (VIVA), constituido por dos componentes: la vigilancia centinela y la vigilancia continua^(32)^. La vigilancia continua pasó a ser obligatoria a partir de 2011, mediante la inclusión de la violencia en la lista de agravios de notificación obligatoria^(33)^, debiendo ser registrada en el SINAN por profesionales de la salud de los servicios públicos y privados. La notificación debe realizarse en cualquier caso sospechoso o confirmado de violencia doméstica/intrafamiliar, sexual, autolesiva, trata de personas, trabajo esclavo e infantil, tortura, intervención legal y violencia homófoba contra mujeres y hombres de todas las edades. En el caso de la violencia extrafamiliar/comunitaria, solo serán objeto de notificación los actos de violencia contra niños, adolescentes, mujeres, personas mayores, personas con discapacidad, indígenas y la población LGBT^(32)^ (Cuadro 1).

El SINAN ha suplido una laguna en el registro de la «violencia» por parte del sector sanitario, ya que el caso se registra de forma directa y no como consecuencia de un acontecimiento, como la atención médica, la hospitalización o la muerte.

Desde la incorporación de la violencia como agravante de notificación obligatoria, los datos del SINAN se han utilizado ampliamente para el análisis y la caracterización de las personas y los diferentes eventos relacionados con la violencia contra las mujeres. A pesar de los esfuerzos por consolidar y ampliar las notificaciones en todo el territorio nacional^(6)^, la subnotificación sigue siendo un reto para los servicios de salud en todas las unidades federativas de Brasil^(34)^.

Teniendo en cuenta las características de los SIS presentadas, se observa que estos sistemas fueron concebidos y organizados para atender demandas específicas, como, por ejemplo, el registro de un evento/agravio o procedimiento y las formas de funcionamiento propias del SUS. A pesar de las limitaciones en términos de cobertura, exhaustividad y calidad de los datos, así como de la falta de interoperabilidad entre los sistemas, sus datos se han utilizado continuamente para la elaboración de diagnósticos relacionados con la violencia contra las mujeres. En este sentido, han desempeñado un papel fundamental en la visibilización del problema y en la planificación de políticas destinadas a mejorar las medidas de lucha contra la violencia.

## EL PAPEL DEL *LINKAGE EN* LA BÚSQUEDA Y DETECCIÓN DE LA VIOLENCIA CONTRA LAS MUJERES

Dado que los SIS están diseñados y estructurados con fines específicos, ya sea para la vigilancia, la atención y la gestión en salud, a menudo de forma independiente, se pueden encontrar redundancias, discrepancias o complementariedades en los datos entre los diferentes sistemas. Si bien esta diversidad de información es beneficiosa, por otro lado, se requiere una inversión en análisis integrados que involucren las diferentes bases de datos.

Debido a la ausencia de un identificador único y a la falta de interoperabilidad entre los SIS de base nacional^(35)^ abordados en este artículo, las áreas de investigación y gestión han recurrido a la relación entre bases de datos (*record linkage*) como estrategia para integrar la información y obtener una visión más amplia de los casos de violencia contra las mujeres. El uso de técnicas de relación de registros puede ayudar a identificar patrones, comprender más a fondo la dinámica de la violencia y proporcionar pruebas importantes para el desarrollo de políticas e intervenciones eficaces.

El *linkage* puede definirse como un método de búsqueda de pares idénticos en una misma base o en bases de datos diferentes^(36,37)^. A partir de esta técnica es posible encontrar pares de registros que se refieren a una misma persona, así como mejorar la completitud y la consistencia de los datos^(36,37)^.

La ausencia de un identificador único y coherente que permita localizar al mismo individuo en diferentes bases de datos^(4)^, como el Registro de Personas Físicas (CPF en la sigla en portugués), por ejemplo, implica una mayor complejidad, ya que requiere el uso de dos o más variables clave, como el nombre y la fecha de nacimiento. Sin embargo, en estos campos pueden producirse errores de mecanografía, uso de abreviaturas o valores faltantes, lo que afectará a la calidad del resultado final del *linkage* ^(35,38)^. En el caso del enfoque determinista, en el que la concordancia debe ser total para la formación de pares, pequeñas diferencias en los nombres o en la fecha de nacimiento darían lugar a que no se encontraran los registros y a la consiguiente pérdida de pares verdaderos^(37)^. En el enfoque probabilístico, en cambio, es necesario determinar un punto de corte para evaluar la similitud de los pares, buscando siempre reducir los falsos positivos y los falsos negativos.

Diversos estudios han utilizado las técnicas de *linkage* para comprender las repercusiones de la violencia en la vida de las mujeres, ampliar la detección de casos y evaluar los riesgos de desenlaces evitables. En Estados Unidos, Cerel et al.^(39)^ utilizaron el *linkage* para analizar la relación entre las muertes violentas y las admisiones previas en los servicios de emergencia por lesiones derivadas de causas externas. La técnica también se utilizó para localizar las muertes maternas asociadas a muertes violentas en Carolina del Norte (EE.UU.)^(40)^. En Brasil, ya se ha aplicado el *linkage* para analizar las muertes por agresión^(41)^, la violencia sexual y sus repercusiones posteriores en el embarazo y el parto^(42)^, mujeres con denuncias de violencia por parte de su pareja íntima y muerte por cualquier causa básica^(43)^, riesgo de muerte en mujeres con denuncias de violencia interpersonal^(44)^ y mortalidad de mujeres con denuncias de violencia durante el embarazo^(45)^.

La integración de los sistemas de información en salud se considera incluso una estrategia importante de la Política Nacional de Vigilancia en Salud (PNVS), en el sentido de contribuir al perfeccionamiento y la consolidación de la gestión de la vigilancia en salud, especialmente en las actividades de planificación, seguimiento y evaluación de las acciones de vigilancia, en el momento oportuno.

Para la vigilancia de la violencia, la vinculación con diferentes SIS contribuye a la producción de información más sólida, a una mejor comprensión de las trayectorias de las mujeres en los servicios de salud, a la identificación de los determinantes de la violencia y del agravamiento de los casos, incluyendo la identificación de posibles perfiles de riesgo de feminicidio. Este procedimiento también permite analizar los procesos de trabajo desarrollados en los servicios relacionados con la atención y la derivación de los casos, promoviendo la identificación de obstáculos y pérdidas de oportunidades del sector de la salud en el abordaje de la violencia.

La relación entre las bases de datos se ha considerado estratégica para la elaboración de políticas públicas en Brasil. Algunos autores sugieren que este tipo de relación se realice periódicamente, incluyendo diversas bases de datos de salud, así como de otras áreas, como la seguridad pública, la justicia y la asistencia social^(41,43,46)^. Sin embargo, al realizar la relación entre las bases de datos, es imperativo tener en cuenta las disposiciones de la Ley General de Protección de Datos (LGPD), que establece directrices estrictas para el tratamiento y el intercambio de datos personales, con el fin de proteger la privacidad y la seguridad de la información de los ciudadanos. Además, la realización del *linkage* no es una actividad sencilla, ya que requiere personal cualificado, acceso a los datos y equipos que permitan el tratamiento de grandes bases de datos.

## PERSPECTIVAS PARA EL FUTURO

Más allá de la falta de interoperabilidad entre los SIS, los análisis del contexto actual del SINASC, SINAN y SIM apuntan a importantes limitaciones, como la falta de estandarización entre campos, la existencia de versiones *off-line*, un lenguaje obsoleto y una baja capacidad de adaptación a los cambios^(47)^.

Con el fin de superar estos problemas, el Ministerio de Salud lanzó el Programa e-SUS Línea de la Vida, cuya implementación está prevista para 2025. Se espera que esta iniciativa sea la única fuente fiable de datos sobre nacimientos, notificaciones y defunciones^(35)^.

Esta propuesta representa un cambio en el enfoque de los SIS, al dirigir la mirada hacia el individuo y no hacia la enfermedad/agravio^(35)^, lo que traerá beneficios para el campo de los estudios y la vigilancia de la violencia. Se espera que con los datos integrados sea posible profundizar en la comprensión de los impactos de la violencia en la salud, ampliar la detección de este evento, actuar en el momento oportuno y contribuir a las acciones de vigilancia y a la realización de investigaciones científicas^(35)^.

Entre las principales potencialidades del Programa destacan: la estandarización de los datos, a partir de la definición de un conjunto mínimo de datos (CMD); la adopción de terminologías estandarizadas; la emisión de declaraciones electrónicas; el envío inmediato de los datos introducidos; las actualizaciones realizadas en tiempo oportuno; y la producción de informes automatizados^(35,47)^.

Sin embargo, es fundamental reconocer que estos sistemas no son solo herramientas esenciales para la gestión de las políticas públicas, sino que también son producto de la interacción continua entre los avances de la tecnología de la información y el contexto organizativo y político. Por lo tanto, se espera que la implementación del programa cobre relevancia en la agenda pública y que se realicen inversiones continuas para garantizar su concretización.

## CONSIDERACIONES FINALES

Los cinco SIS de ámbito nacional abordados en este artículo permiten el registro explícito de casos de violencia, como las notificaciones de violencia interpersonal o autoproducida en el SINAN; las muertes por agresión autoproducida, interpersonal o de intención indeterminada en el SIM; los diagnósticos, principales y secundarios, en las hospitalizaciones y en los procedimientos ambulatorios, en el SIH y el SIA; y los casos de violación de personas vulnerables en niñas menores de 14 años con hijos nacidos vivos en el SINASC.

Sin embargo, la complejidad de la violencia contra las mujeres exige un enfoque integrado de los datos, lo que se ha logrado mediante la realización de *enlaces* entre los diferentes SIS. A pesar de ser la estrategia posible en la actualidad, cabe destacar que existen limitaciones en sus resultados, derivadas de la exhaustividad y la calidad de los datos originales.

El lanzamiento del Programa e-SUS Línea de la Vida por parte del Ministerio de Salud representa un avance importante en la búsqueda de la integración de los sistemas de información en salud. Al dirigir el foco hacia el individuo y no solo hacia la enfermedad/agravio, el programa pretende superar los problemas de fragmentación y falta de estandarización, convirtiéndose en una fuente única y confiable de datos de nacimientos, notificaciones y defunciones. Este enfoque supone un cambio de paradigma en la vigilancia en salud, en el que el individuo pasa a ser el centro, un cambio que sigue la tendencia, no reciente, del modelo de atención y cuidado previsto en las políticas públicas de salud en Brasil. Así, se espera que esta perspectiva de vigilancia en salud pueda sumar esfuerzos a la necesidad de ofrecer a las mujeres en situación de violencia una atención integral y humanizada, articulando la detección, la atención y la derivación adecuados de los casos y garantizando una mirada sensible a sus especificidades.

Por otra parte, es importante destacar que solo los registros ya existentes, como se ha señalado al principio de esta sección, permitirían una actuación más eficaz en el tratamiento de los casos de violencia contra las mujeres en el sector sanitario, por ejemplo, a partir de la producción de información y su retroalimentación a la atención primaria y/o especializada. También permitiría el seguimiento de los casos utilizando los sistemas que registran los procedimientos, por ejemplo, en el caso de la violencia sexual.

Así, se espera que los cambios en curso, importantes y deseables, pero eventualmente lentos, no anulen el potencial que tienen los sistemas, aunque sea de forma individual, para proporcionar datos e información valiosa para hacer frente a la violencia contra las mujeres, un fenómeno para el que se necesitan urgentemente medidas más eficaces por parte del Estado brasileño.

## Footnotes

1 En Brasil, el feminicidio cobró relevancia en el ámbito de las políticas públicas cuando, en 2015, entró en vigor la Ley n.º 13.104, que lo definió como el homicidio cometido contra una mujer por razones de su condición de mujer, es decir, cuando implica violencia doméstica y familiar o menosprecio o discriminación por razón de su condición de mujer.
